# Revealing how an adenylate cyclase toxin uses bait and switch tactics in its activation

**DOI:** 10.1371/journal.pbio.2005356

**Published:** 2018-02-27

**Authors:** Natosha L. Finley

**Affiliations:** 1 Department of Microbiology, Miami University, Oxford, Ohio, United States of America; 2 Cell, Molecular, and Structural Biology Program, Miami University, Oxford, Ohio, United States of America

## Abstract

Dissecting how bacterial pathogens escape immune destruction and cause respiratory infections in humans is a work in progress. One tactic employed by microbes is to use bacterial adenylate cyclase toxins (ACTs) to disarm immune cells and disrupt cellular signaling in host cells, which facilitates the infection process. Several clinically significant pathogens, such as *Bacillus anthracis* and *Bordetella pertussis*, have ACTs that are stimulated by an activator protein in human cells. Research has shown that these bacterial ACTs have evolved distinct ways of controlling their activities, but our understanding of how the *B*. *pertussis* ACT does this is limited. In a recent study, O’Brien and colleagues provide new and exciting evidence demonstrating that the regulation of *B*. *pertussis* ACT involves conformational switching between flexible and rigid states, which is triggered upon binding the host activator protein. This study increases our knowledge of how bacterial ACTs are unique enzymes, representing a potentially novel class of drug targets that may open new pathways to combat reemerging infectious diseases.

## The reemerging threat of *B*. *pertussis*

Whooping cough (also known as pertussis) is an infectious respiratory disease found in adults and adolescents, but which is most harmful and even fatal to infants and young children. The respiratory pathogen *B*. *pertussis* uses a number of damaging enzymes, including an adenylate cyclase toxin (ACT) known as CyaA, to facilitate causing whooping cough in humans. Historically, whooping cough infections killed infants around the globe, but modern medicine reduces the impact of this deadly disease using a combination of antibiotics and immunization. At present, pertussis infections are increasing, despite being a vaccine-preventable disease and the emergence of antibiotic resistant strains is a global public health concern [1].

## Bacterial adenylate cyclase toxins: An incomplete picture

The bacterial ACTs convert adenosine triphosphate (ATP) to cyclic adenosine monophosphate (cAMP), allowing for its high intracellular accumulation to pathogenic levels. There are different bacterial ACTs—edema factor (EF) released by *Bacillus anthracis*, CyaA made by *B*. *pertussis*, and ExoY, which is produced by *Pseudomonas aeruginosa*. Both EF and CyaA are calmodulin (CaM)-dependent, while ExoY is stimulated by actin, the significance of which is reviewed elsewhere [2]. EF entry into the host cell requires endocytosis and translocation through a pore formed by an accessory protein known as protective antigen. However, CyaA is composed of a C-terminal pore-forming hemolysin domain that transports the N-terminal adenylate cyclase domain of CyaA (CyaA-ACD) into the host cell for CaM-dependent activation. The early establishment of *B*. *pertussis* infections is facilitated by CyaA, which disrupts the action of immune cells, allowing the microbe to escape destruction [3,4]. CyaA-deficient *B*. *pertussis* strains are less virulent [5], and antibody-mediated neutralization of CyaA protects against infection [6]. In order to facilitate bacterial colonization during infection, ACTs must compete with other effector proteins such as host cell adenylate cyclases (ACs) for CaM association. A persistent question in the study of *B*. *pertussis* pathogenesis has been: how does CyaA selectively target host cell CaM?

## How ACTs impact the host cell activator protein calmodulin

In this primer, we focus on how CaM-dependent bacterial ACTs work by highlighting the unique mechanism by which CyaA activity is stimulated by the host activator CaM. This ubiquitous eukaryotic protein is a globular metal-sensing protein, which is important in a variety biological functions such as cellular signaling, metabolism, memory formation, and channel activation in host cells [7]. CaM is composed on N-terminal and C-terminal domains that bind intracellular Ca^2+^ and Mg^2+^, with one of its primary physiological roles being Ca^2+^-regulated enzyme stimulation. One normal physiological function of CaM is to activate host ACs through moderate affinity interactions, leading to the increased production of intracellular cAMP, a critical secondary messenger molecule in the cell. Interestingly, bacterial ACTs such as CyaA remain largely inactivated until they gain access to the host cell, where they wreak havoc by disrupting cAMP levels through CaM-dependent activation [8]. Much speculation exists as to how bacterial ACTs exert their selective effects during host infection. There is compelling evidence that CyaA exploits its high-affinity interaction with CaM to commandeer this metal-sensing protein, and the physiological consequences are likely to be disruption of cellular responses to Ca^2+^ fluctuations [9]. To this end, other urgent questions emerge, such as how does the activation of CyaA-ACD differ from other CaM-dependent bacterial ACTs and what is the role of metal binding in these processes?

## Bacteria ACTs have distinct structural and functional mechanisms

**Fig 1:**
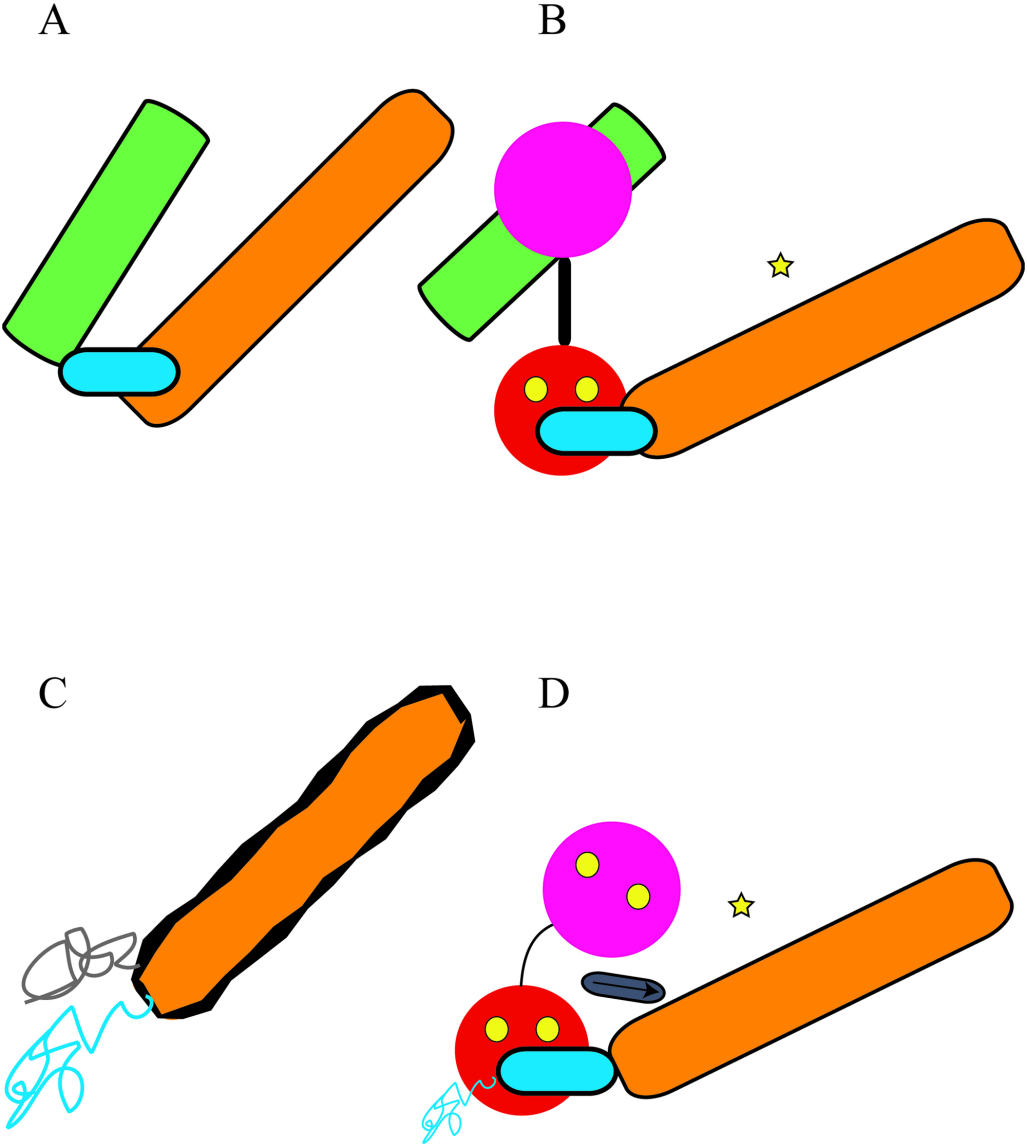
Model depicting the dispositions of structural elements important in the CaM-dependent activation of two bacterial ACTs. (A) In the absence of CaM, the helical domain of EF (green rectangle) limits the accessibility of the catalytic core (orange rectangle), which contains the CaM-binding determinant (cyan ellipse). (B) When CaM (depicted by a pink sphere representing the N-terminal domain and a red sphere denoting the C-terminal domain) binds in the presence of 2–4 Ca^2+^ ions (yellow spheres), the helical domain reorients, exposing the catalytic site (yellow star) of EF. (C) Surprisingly, two key structural elements of CyaA-ACD are disordered in the absence of CaM (gray and cyan traces), while the catalytic core (orange) is unstably folded (flexibility is depicted by wavy black outline). (D) CyaA-ACD, which lacks a helical domain, is inactive in the absence of CaM, presumably due to the lack of a stable catalytic site. In the presence of 4Ca^2+^-CaM, the domain involved in C-terminal CaM-binding (cyan ellipse) and another key region (gray ellipse with black arrow) become stably folded with increased rigidity. Furthermore, the catalytic core (orange rectangle with solid black line) is stabilized by CaM-binding and readily converts ATP to cAMP. ACT, adenylate cyclase toxin; ATP, adenosine triphosphate; CaM, calmodulin; cAMP, cyclic adenosine monophosphate; CyaA-ACD, adenylate cyclase domain of CyaA; EF, edema factor.

There is little conservation at the amino acid level between bacterial ACTs; therefore, EF and CyaA are not structural homologs. However, they do share functional similarities. A detailed picture of EF structural transitions exists due in part to the availability of experimentally determined structures and biophysical characterization of CaM/EF complexes [10,11]. The activation of EF is physically blocked by the presence of the helical domain (Fig 1A and 1B). Upon CaM interaction and in the presence of high Ca^2+^concentration, the helical domain is rotated, exposing the ATP-binding pocket, which ultimately results in EF stimulation.

In contrast, CyaA lacks a comparable helical domain (Fig 1C and 1D) and is activated by CaM at lower Ca^2+^ [9]. The C-terminal domain of CaM is critical in the activation of CyaA-ACD [12], but the importance of N-terminal CaM and the contributions of key regions in CyaA-ACD remain unclear. These unanswered questions compel researchers to postulate that CyaA has evolved a unique mechanism for cAMP production, possibly through the exploitation of dynamics and allosteric mechanisms not previously identified in bacterial ACTs, but the molecular details of this switching mechanism remain to be determined. Mounting evidence suggests the possibility that conformational “shape-shifting” underlies the distinct regulatory mechanism of CaM-dependent regulation of CyaA. With this emerging concept, the following line of inquiry ensues—is the enzymatic output of CyaA susceptible to fine-tuning through changes in protein global conformation and dynamics?

## The role of dynamic conformational changes in ACTs function

Unfortunately, the absence of high-resolution structures of CyaA (both free and bound to intact CaM) limits the understanding of how CaM-binding induces activation. The conformation of free CyaA is known to be heterogeneous, and structural studies performed using isolated CyaA-ACD do not mitigate this intrinsic property of the toxin, as it too remains recalcitrant to structure determination. Aggregation of CyaA in the absence of CaM is modulated by sample conditions and posttranslational modifications allowing it to be folded into monodisperse, functional conformers [13,14]. In the presence of CaM, isolated CyaA-ACD forms stable complexes that are monodisperse and functionally active [15,16], which is advantageous in the structural study of this system.

Recombinant CyaA-ACD engages CaM tightly (nM affinity), with the C-terminal CaM contributing most of the binding energy for this interaction, while N-terminal CaM association is much weaker [9,17], suggesting that conformational fluctuations play a significant role in CaM-dependent CyaA-ACD function. Guo and colleagues used biochemical, biophysical, and computational approaches to show that interaction occurs between N-terminal CaM and CyaA-ACD [18]. Domain flexibility and metal-sensing properties of CaM are affected by CyaA-ACD [16,19–21]. Hydrodynamic changes (which are indicative of changes in global shape [15]) and long range structural rearrangements [19,22] have been reported in CaM/CyaA-ACD complexes, also pointing to the importance of conformational plasticity in this *B*. *pertussis* virulence factor. Targeted mutations reduce conformational stability and activation, providing further evidence that both dynamics and unique structural determinants are crucial regulators controlling CaM-dependent stimulation of CyaA-ACD [12,16,18,19,21–23]. Taken together, these findings imply that conformational switching, particularly in CyaA-ACD, is modulated in part by the formation of stabilizing interactions with CaM and is crucial to enzymatic activation. With this knowledge, light is cast on the potential existence of interplay between conformational disorder and activation state in CaM-dependent CyaA-ACD. The prevailing question now becomes: how do both dynamic motions and stabilizing intermolecular contacts work in concert to regulate CaM-dependent activation of CyaA-ACD?

In a recent study published in *PLOS Biology*, O’Brien and colleagues report on the importance of shape-shifting in CyaA-ACD’s CaM-dependent catalytic function [24]. New structural information derived from this study demonstrates how a disorder-to-order conformational transition in CyaA-ACD is modulated by CaM-binding and long range allosteric effects influence CaM-dependent activation of CyaA-ACD. The authors utilize synergistic biophysical techniques that are useful in the structural characterization of biomolecules in solution to demonstrate that CaM interaction induces a transition from a disordered to ordered state in CyaA. These methodologies also permit the examination of structural changes occurring in enzyme systems that have a combination of low- and high-affinity structural binding determinants controlling the recruitment of the activator protein, a key feature that has been reported for CaM/CyaA-ACD complex. Their experimental design seeks to discriminate between induced folding events, conformational rearrangements, and long-range allosteric effects produced by CaM-binding to CyaA-ACD. The most pressing issue is then defining what CaM-binding does to the structure of CyaA-ACD and whether protein dynamics function in this process.

Surprisingly, the region of CyaA-ACD that is primarily responsible for CaM engagement is only transiently folded in the absence of CaM. Moreover, a disorder-to-order transition occurs upon CaM association, which stabilizes that structural unit of CyaA-ACD. In addition to localized structural changes, the authors report that binding mediated by the C-terminal domain of CaM induces allosteric remodeling of the catalytic pocket of CyaA-ACD while preserving flexibility in the catalytic loop. In this way, the catalytic pocket is held steady when CyaA-ACD is CaM-bound, while dynamic motion in the catalytic loop facilitates the rapid conversion of ATP to cAMP. The authors demonstrate that C-terminal CaM is unequivocally protected from solvent exposure in the presence of CyaA-ACD, further corroborating the formation of a high-affinity complex. However, the conformational effects of CyaA-ACD association on the N-terminal domain of CaM are not as clearly defined. From a global perspective, the complex exists as an extended structure. However, the flexible tether connecting the two lobes of CaM allows for considerable N-terminal domain mobility, even when C-terminal CaM is bound to CyaA-ACD. As a consequence, the N-terminal domain is free to sample conformational space without experiencing dramatic conformation changes upon CyaA-ACD association. In summary, CyaA utilizes an unstructured “bait” to lure CaM, forming an activation complex. Subsequently, CyaA “switches” from a disordered-to-ordered state, striking an adaptable balance between rigidity and flexibility, which provides a platform for fine-tuning CaM-dependent activation.

## A new biological paradigm?

While there are examples of CaM-binding inducing structural order in other enzymes, such as calcineurin [25], the findings reported in *PLOS Biology* point toward the likelihood that *B*. *pertussis* ACT exhibits a previously uncharacterized mechanism for CaM-induced enzymatic stimulation that functions on a global scale. As such, the work of O’Brien and colleagues provides a springboard for continued research and presents a number of thought-provoking questions to explore. For example, what is the role of induced structural changes that occur in regions outside of the primary CaM-binding determinant? Other studies have shown that mutation in these regions of CyaA-ACD impacts conformation and activation, suggesting sites of importance in CaM-dependent stimulation of this enzyme [16,18]. O’Brien reports that the solvent accessibility of CaM is impacted by both intact CyaA and CyaA-ACD with some minor differences. It is tempting to speculate on the significance of any differences in induced conformational changes in CaM outside of the primary C-terminal binding sites. CyaA-ACD association does not mediate large conformational changes in N-terminal CaM, which is in good agreement with other studies. Perhaps we can take another step toward elucidating the role of N-terminal CaM in CyaA-ACD activation by exploring the range of detection that the solvent accessibility experiments have in capturing transient interactions. Future experiments should test the “crowding” hypothesis by continuing to explore the contribution of N-terminal CaM in CyaA-ACD stimulation using distance measurements. Furthermore, elucidating the novel mechanism by which intact CaM activates CyaA might permit the design of highly specific compounds targeting this interaction. The possibility of designer drugs targeting only CyaA-ACD that do not disrupt mammalian AC interaction with CaM offers promise in the development of antitoxin therapies that may prevent or treat pertussis in the age of evolving antibiotic resistance.

## Abbreviations

AC: adenylate cyclase
ACT: adenylate cyclase toxin
ATP: adenosine triphosphate
CaM: calmodulin
cAMP: cyclic adenosine monophosphate
CyaA-ACD: adenylate cyclase domain of CyaA
EF: edema factor
